# The Nanotechnology-Based Approaches against Kirsten Rat Sarcoma-Mutated Cancers

**DOI:** 10.3390/pharmaceutics15061686

**Published:** 2023-06-08

**Authors:** Fernanda Andrade, Júlia German-Cortés, Sara Montero, Pilar Carcavilla, Diego Baranda-Martínez-Abascal, Marc Moltó-Abad, Joaquín Seras-Franzoso, Zamira Vanessa Díaz-Riascos, Diana Rafael, Ibane Abasolo

**Affiliations:** 1 Clinical Biochemistry, Drug Delivery and Therapy Group (CB-DDT), Vall d’Hebron Institut of Research (VHIR), Vall d’Hebron University Hospital, Vall d’Hebron Barcelona Hospital Campus, 08035 Barcelona, Spain; fernanda.silva@vhir.org (F.A.); julia.german@vhir.org (J.G.-C.); sara.montero412@gmail.com (S.M.); pilarcarcavilla@gmail.com (P.C.); diego.baranda@vhir.org (D.B.-M.-A.); marc.molto@vhir.org (M.M.-A.); joaquin.seras@vhir.org (J.S.-F.); vanessa.diaz@vhir.org (Z.V.D.-R.); 2 Centro de Investigación Biomédica en Red de Bioingenería, Biomateriales y Nanomedicina (CIBER-BBN), Instituto De Salud Carlos III, 08035 Barcelona, Spain; 3 Departament de Farmàcia i Tecnologia Farmacèutica i Fisicoquímica, Facultat de Farmàcia i Ciències de l’Alimentació, Universitat de Barcelona (UB), 08028 Barcelona, Spain; 4 Functional Validation & Preclinical Research (FVPR)/U20 ICTS Nanbiosis, Vall d’Hebron Institut de Recerca (VHIR), Universitat Autònoma de Barcelona (UAB), 08035 Barcelona, Spain; 5 Department of Genetics and Microbiology, Universitat Autònoma de Barcelona (UAB), 08193 Bellaterra, Spain; 6 Clinical Biochemistry Service, Vall d’Hebron University Hospital, Vall d’Hebron Barcelona Hospital Campus, 08035 Barcelona, Spain

**Keywords:** KRAS, KRAS mutation, nanotechnology, nanomedicine, cancer treatment, delivery systems

## Abstract

Kirsten rat sarcoma (KRAS) is a small GTPase which acts as a molecular switch to regulate several cell biological processes including cell survival, proliferation, and differentiation. Alterations in KRAS have been found in 25% of all human cancers, with pancreatic cancer (90%), colorectal cancer (45%), and lung cancer (35%) being the types of cancer with the highest mutation rates. KRAS oncogenic mutations are not only responsible for malignant cell transformation and tumor development but also related to poor prognosis, low survival rate, and resistance to chemotherapy. Although different strategies have been developed to specifically target this oncoprotein over the last few decades, almost all of them have failed, relying on the current therapeutic solutions to target proteins involved in the KRAS pathway using chemical or gene therapy. Nanomedicine can certainly bring a solution for the lack of specificity and effectiveness of anti-KRAS therapy. Therefore, nanoparticles of different natures are being developed to improve the therapeutic index of drugs, genetic material, and/or biomolecules and to allow their delivery specifically into the cells of interest. The present work aims to summarize the most recent advances related to the use of nanotechnology for the development of new therapeutic strategies against KRAS-mutated cancers.

## 1. Introduction

KRAS is one of the most studied and challenging targets in cancer research, mainly due to the enormous difficulty of treating KRAS-mutated cancers. For several years, KRAS was considered an untargetable and undruggable target, and KRAS-mutated cancer was considered untreatable. Recently, a few therapeutic strategies demonstrated efficacy in targeting this molecule. Since this is a field in clear expansion, in the present work, the most recent studies aiming to treat KRAS-mutated cancers using nanotechnology-based approaches able to target KRAS will be presented.

### KRAS in Cancer

**Figure 1:**
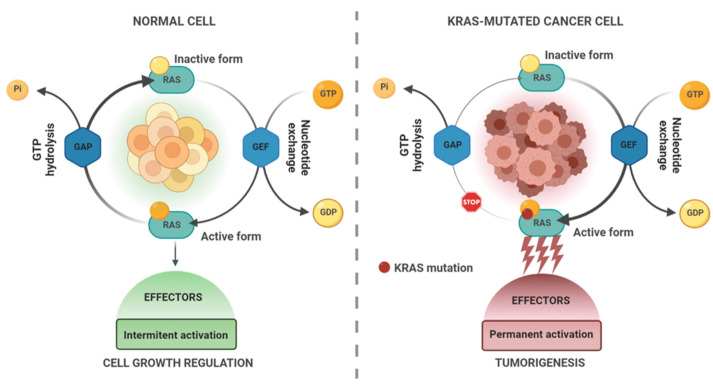
Schematic of the KRAS mutations mechanism and KRAS targeting therapies. Created in BioRender.com. Abbreviations: GAP—GTPase-activating protein, GDP—guanosine diphosphate, GEF—guanine nucleotide exchange factor, GTP—guanosine triphosphate, Pi—inorganic phosphate.

KRAS is a small guanosine triphosphate (GTP)-binding protein (21 kDa) belonging to a large group of RAS-like GTPases also known as RAS superfamily proteins. KRAS works as a molecular switch in the guanosine diphosphate-GTP (GDP-GTP) cycle, transducing signals via a simple binary on–off mechanism changing from an inactive GDP-bound to an active GTP-bound [1,2] (Figure 1). KRAS regulation is mediated by guanine nucleotide exchange factors (GEFs; e.g., SOS1), which promote nucleotide exchange and produce the RAS-GTP active state, and GTPase-activating proteins (GAPs; e.g., neurofibromin) that stimulate the hydrolysis of the bound GTP, leading to its inactive RAS-GDP form. Under normal physiological conditions, KRAS is predominantly inactive. In response to extracellular stimuli, such as growth factors or GEF activation, GTP binds to KRAS and undergoes conformational changes, which activates the protein. Activated KRAS-GTP binds and activates a spectrum of catalytically downstream effector proteins that regulate important cellular processes and a diversity of intracellular signaling networks and pathways, including the mitogen-activated protein kinase (MAPK) pathway [3,4,5], PI3K/AKT/mTOR signaling cascade [6,7], and the Ral-GEF pathway [8,9]. These pathways are involved in cell survival, proliferation, differentiation, migration, and apoptosis processes.

Oncogenic mutations in KRAS protein, presented in approximately 90% of pancreatic cancers, 45% of colon cancers, and 35% of lung cancers, impair GAP-stimulated GTP hydrolysis activity, which hampers the protein in switching between active and inactive states, rendering RAS in constitutively active GTP-bound status [2,10,11] (Figure 1). This permanent activation causes an overstimulation of downstream cascades, resulting in a promotion of tumorigenesis by mechanisms such as increased proliferation, apoptosis suppression, migration, altered metabolism, changes in the tumor microenvironment, dysregulation of membrane vesicle trafficking and cytoskeleton organization, immune system response evasion, and metastasis development [1,12,13,14].

Cancer-associated RAS genes are characterized by single-base missense mutations, which are predominantly found at codons glycine-12 (G12), glycine-13 (G13), and glutamine-61 (Q61) [2,15]. While KRAS-G12 mutations are frequent in pancreatic (91% of KRAS-mutated cases), colorectal (CRC) (68%), and lung (85%) cancers, KRAS-G13 mutations are common in gastrointestinal cancers (20% in CRC), and KRAS-Q61 alterations are common in human melanomas (85%) [9,16]. Nonetheless, although 98% of all RAS mutations are located at these canonical codons, other infrequent mutations at non-canonical codons, such as 19, 22, 59, 117, and 146, have also been described, showing the biological complexity related to these oncogenes [12,17].

KRAS mutations negatively affect the patient’s prognosis, survival, and response to chemotherapy [1,15], being associated with resistance to chemotherapy. For these reasons, KRAS is commonly used as a biomarker for treatment selection. Different RAS mutations may display distinctive functional and biological outcomes depending on (i) the tissue of origin, (ii) the type of RAS isoform, (iii) the mutated codon, (iv) the amino acid substitutions, and (v) the presence of post-transduction alterations; accordingly, RAS proteins are able to anchor in different subcellular membranes and activate different signaling pathways. Despite these small differences, the negative impact on patients’ clinical outcomes is similar. Thus, to achieve effective treatment in oncologic patients that present a KRAS mutation, it is of utmost importance to develop therapeutic strategies targeting the KRAS protein.

## 2. Actual Therapeutic Strategies against KRAS

Since the discovery of mutations in the KRAS gene in the early 1980s, many researchers have worked on targeting this protein to effectively treat cancer. Unfortunately, this has shown to be a hard task with more failures than successes, which could be explained by the molecular complexity and small size of the KRAS protein and its smooth structure missing deep pockets where drugs can be bound [18]. Moreover, KRAS possesses considerable post-transcriptional modifications, binding tightly to GTP, making the blockage of its hyperactivation difficult in the case of KRAS-mutated cancers [19]. For these reasons, KRAS was considered an “undruggable” target for many years. The majority of strategies for treating KRAS-mutated cancers are based on indirect therapies targeting, for example, the nucleotide exchange, membrane receptors, metabolic rewiring, and other effectors (RAF, MEK, PI3K, mTOR, FAK, EGFR, etc.) of signaling pathways in which KRAS is involved such as MAPK or EGFR [20,21,22].

Nonetheless, with a better understanding of the KRAS protein structure and dynamics observed in the last 10 years, some light appears at the end of the tunnel for treating KRAS-mutated cancers, since some compounds targeting specific KRAS mutant forms have been developed and enrolled in clinical trials [18,19,23]. These KRAS-targeted therapies include AMG510 (sotorasib) and MRTX849 (adagrasib).

Sotorasib (Lumakras/Lumykras, Amgen, Inc., Thousand Oaks, CA, USA) was the first (2021) compound approved by both the FDA and EMA (based on CodeBreaK 100 clinical trial NCT03600883) for treating KRAS-mutated (G12C) locally advanced or metastatic non-small cell lung cancer (NSCLC) [24,25]. Sotorasib binds covalently and irreversibly to a cysteine, leading to the blockage of the protein and its biological activity [24,26]. On December 2022, adagrasib (Krazati, Mirati Therapeutics, Inc., San Diego, CA, USA) was granted accelerated approval by the FDA (based on KRYSTAL-1 clinical trial NCT03785249) [27]. Like sotorasib, adagrasib binds irreversibly to G12C-mutated KRAS, being approved to treat locally advanced and metastatic NSCLC [28]. Other KRAS G12C form inhibitors have been developed, including MRTX1257 [29], ARS-853 [30], ARS-1620 [31], LLK-10 [32], and analogs [33,34] of the last two.

Despite the encouraging results obtained so far, these strategies focus on the G12C mutation, and the other KRAS-mutated forms remain untargeted. For this reason, compounds targeting other KRAS forms and pan-KRAS inhibitors are being developed. KRpep-2d [35] and KS-58 [36,37] were developed to target KRAS G12D mutation, while 12VC1 [38] is able to selectively recognize the active state of both G12V and G12C forms. VS-6766 (Avutometinib) is a dual RAF–MEK inhibitor that showed good therapeutic activity against KRAS G12V mutation and is under clinical evaluation (NCT03875820, Phase I, NCT04625270, Phase II) for different types of KRAS-mutated cancers [39].

Examples of pan-KRAS inhibitors are BBP-454 (BridgeBio Pharma, preclinical evaluation), BI 1701963 (Boehringer Ingelheim, NCT04111458, Phase I) [40], and AZD4785 (AstraZeneca, Inc., Cambridge, UK, NCT03101839, Phase I) [41]. AZD4785 is a high-affinity constrained ethyl-containing therapeutic antisense oligonucleotide (ASO) proposed to target all KRAS isoforms [42], while BI 1701963 interferes with KRAS binding to SOS1, a guanine nucleotide exchange factor essential in activating KRAS [40,43].

The use of non-small molecules such as antibodies, peptides, or oligonucleotides for targeted therapies has also been proposed. However, the difficulty for such molecules to reach the intracellular compartment in their active form is the major handicap of using and translating such strategies from the bench to the bedside.

It is worth mentioning the development of advanced cell therapies and cancer vaccines targeting mutated KRAS that are also under development and clinical assessment [44]. For example, peripheral blood lymphocytes with modified mTCR targeting KRAS G12D (NCT03745326, Phase I/II) and KRAS G12V (NCT03190941, Phase I/II) mutations are under clinic evaluation for rectal and pancreatic cancer, respectively [41,44]. In addition, mutant KRAS G12V-specific TCR transduced T cells were developed for pancreatic cancer treatment (NCT04146298, Phase I/II) [41]. Regarding vaccines, an mRNA-based cancer vaccine (V941) targeting KRAS mutations (G12D, G12V, G12C, and G13D) is under clinical trials to treat solid tumors (NCT03948763, Phase I). A KRAS peptide vaccine (NCT04117087, Phase I), and a dendritic cell-based vaccine targeting the KRAS G12C, G12D, G12R, and G12V forms (NCT03592888, Phase I) are also under development for CRC and pancreatic cancer treatment [41]. Other advanced therapies, in this case using cell derivative nanoparticles that are under clinical evaluation (Phase I), comprise iExosomes, extracellular vesicles loaded with siRNA for the specific inhibition of KRAS G12C in pancreatic cancer (NCT03608631) [45].

Notwithstanding the promising results obtained with the proposed strategies, unfortunately, some compounds on the pipeline are failing to reach the desired endpoints during clinical assessment. This is the example of AZD4785 which was discontinued after completing Phase I clinical assessment due to insufficient KRAS-lowering capacity, according to AstraZeneca [37]. In addition, JNJ-74699157 (Janssen) targeting KRAS G12C mutation was discontinued after Phase I studies (NCT04006301) due to an unfavorable safety profile [46]. Moreover, despite the recent approval, the development of resistance to the KRAS G12C inhibitors has already been reported, limiting the therapeutic efficacy and clinical application of such drugs to treat KRAS-mutated cancers [47,48]. Thus, it is necessary to develop innovative formulations able to surpass the limitations of the therapies currently approved and under development.

## 3. The Importance of Nanotechnology

Conventional cancer therapies present several limitations due to the lack of drug specificity to the tumor site, insufficient penetration capacity, low solubility, and the development of drug resistance, among others. Most of these drawbacks can be overcome by nano-sized drug delivery systems (nanoDDSs), leading to an improvement in the therapeutic index of drugs [49,50,51].

Generally, nanoDDSs passively target and accumulate at solid tumor sites and inflamed tissues through the enhanced permeability and retention (EPR) effect, which is a result of abnormal tumor angiogenesis. During tumor formation, tumor cells rapidly recruit new blood vessels in order to receive more oxygen, nutrients, and other growth factors. Because of this fast and imperfect angiogenesis, newly formed blood vessels present an immature and discontinuous epithelium, where fenestrations are larger than 100 nm [50,51]. These gaps between tumor endothelial cells allow particles to be extravasated from vessels to the interstitial tumor space. In addition to these structures, tumor tissues are also characterized by the lack of adequate drainage of lymphatic systems, which allows compounds to be retained for longer periods than observed in normal tissue, increasing the therapeutic efficacy [52]. Contrary to tumors, healthy tissues do not exhibit large fenestrations and have functional lymphatics. Therefore, nanoparticles (NPs) will not be able to extravasate into normal tissues, reducing the level of adverse effects [50,52].

Nevertheless, there are a number of factors that prevent the efficient accumulation of nanoDDSs in tumor tissues via the EPR effect at the required therapeutic doses, such as high interstitial fluid pressure in tumors, poor lymphatic drainage, tissue penetration, nonvascular tumor tissue, and liver and spleen accumulation [52]. Concerning the last problem, it is essential to consider the particle’s size, charge, and shape. The optimal NP size is between 10 and 100 nm since particles smaller than 10 nm rapidly undergo renal clearance, and those higher than 100 nm accumulate in the liver and spleen due to their vascular fenestrations (200–500 nm). Regarding the shape, non-spherical NPs, such as cylindrical and needle particles, can accumulate less in the liver, spleen, and kidney and more in the tumor. By contrast, spherical NPs are taken up by the cells more efficiently under fluid flow conditions [51,53]

To further improve the accumulation and retention of NPs in tumors beyond the EPR effect, active targeting can be used. The active targeting strategy consists of decorating the NP surface with different moieties, which interact specifically with biomarkers or receptors overexpressed on the tumor cells. Diverse tumor-targeting ligands have been proved for NP functionalization, involving, among others, monoclonal antibodies, nanobodies, peptides, and carbohydrates [54].

Another challenging factor for applying NPs to the cancer field is their rapid metabolism and clearance from the bloodstream by reticuloendothelial system (RES) cells. To overcome this drawback, the NP surface is coated with an inert hydrophilic polymer; polyethylene glycol (PEG) is mainly used, but others such poly(2-oxazoline) (POx) or poly(zwitterions) are also used. These stealth polymers produce a reduction in opsonization, prevention of aggregation, and steric hindrance to block the binding of RES cells. In this way, the covering with PEG increases the in vivo circulation time of NPs and the probability of reaching and accumulating in tumors [55,56].

Several types of NPs have been explored for cancer treatment, including in the development of new anti-KRAS therapies, such as liposomes, solid lipid nanoparticles, dendrimers, polymeric micelles, polymeric nanoparticles, inorganic particles, and extracellular vesicles [49,57]. The advantages and limitations of each type of nanoplatform are shown in Table 1.

## 4. Nanotechnology-Based Anti-KRAS Therapies: The State of the Art

**Figure 2:**
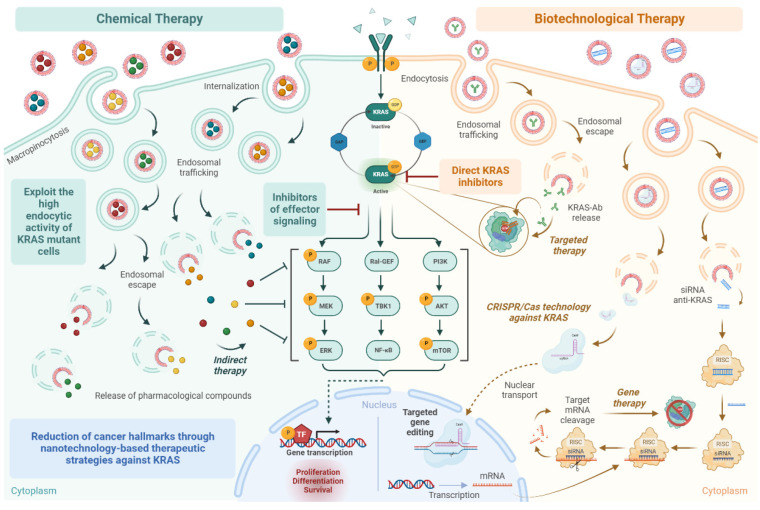
Schematic representation of the different strategies based on nanotechnology for KRAS-mutated cancer treatment. Created in BioRender.com. Abbreviations: AKT—protein kinase B, CRISPR/CAS—clustered regularly interspaced short palindromic repeats/CRISPR associated nuclease, ERK—extracellular signal-regulated kinase, GAP—GTPase-activating protein, GDP—guanosine diphosphate, GEF—guanine nucleotide exchange factor, GTP—guanosine triphosphate, KRAS—Kirsten rat sarcoma viral oncogene homolog, KRAS-Ab—antibody against KRAS, MEK—mitogen-activated protein kinase, mRNA—messenger RNA, mTOR—mammalian target of rapamycin, NF-κB—nuclear factor kappa B, P—phosphate, PI3K—phosphoinositide-3-kinase, RAF—NFKB1 nuclear factor kappa B subunit 1, Ral-GEF—Ras-like small GTPase, RISC—RNA-induced silencing complex, siRNA—small interfering RNA, TBK1—TANK-binding kinase 1, TF—transcription factor.

Different strategies based on nanotechnology to deliver small chemical molecules, as well as biomolecules and advanced therapies, including gene therapy, have been proposed to treat KRAS-mutated cancers (Figure 2). In this section and in Table 2, some examples of nanomedicines that have been developed in the last 5 years will be presented.

### 4.1. Chemical Therapy

As mentioned above, only two small drugs have recently been approved for clinical use, and not much research has been conducted to develop nanoformulations with this type of drug. The majority of studies using nanomedicines to target KRAS-mutated cancers are based on indirect therapies as mentioned in Section 2. For example, regarding metabolic rewiring, KRAS-mutated cancer cells possess a high endocytic activity mainly via micropinocytosis to allow a high intake of nutrients. Based on this, Liu, X, et al. (2019) proposed albumin-based nanoparticles that are taken up by macropinocytosis as a good approach to deliver pharmacological compounds preferentially to KRAS-mutated cells [58]. Similar behavior was also reported by Li, R, et al. (2021) with nanoparticulate albumin-bound paclitaxel [59] and Dou, L, et al. (2022) with β-lapachone albumin nanoparticles [60].

Regarding the downstream effectors, afatinib, an irreversible tyrosine kinase inhibitor, initially approved as first-line treatment of late-stage metastatic NSCLC, was encapsulated into inhaled polylactic-co-glycolic acid (PLGA) nanoparticles [61]. The encapsulation of afatinib improved its efficacy against KRAS-mutated NSCLC cell lines (A549, H460) and its penetration into 3D spheroids. In addition, the aerosol presents appropriate aerodynamic properties for deep lung deposition. Binimetinib is a MEK 1/2 inhibitor that has shown clinical therapeutic efficacy against KRAS-mutated cancers such as acute myeloid leukemia, colorectal cancer, melanoma, and NSCLC, especially when in combination with other drugs [62,63,64]. Binimetinib has a high plasma protein binding and a short half-life, thus being a good candidate for nanoencapsulation. In fact, Bikhezar, F, et al. (2022) efficiently encapsulated binimetinib into polymersomes of poly(butadiene-b-ethylene oxide) block copolymers [65]. Another drug that has been proposed for indirect therapy of KRAS-mutated cancers due to its RAF kinase inhibitory activity is sorafenib, approved for the treatment of advanced hepatocellular carcinoma [66,67]. Due to its poor water solubility and rapid metabolization and clearance, sorafenib has also been encapsulated in different types of nanoparticles, from PLGA/PEG to liposomes, albumin nanoparticles, carbon nanotubes, or polymeric micelles [68,69,70]. In addition, since in KRAS mutant cells, B-raf inhibition activates upstream proteins leading to ERK activation through an alternative pathway, sorafenib should not be used as single-agent therapy in KRAS-mutated cancers [23,71], and nanoparticles offer the possibility for simultaneous delivery of different agents. Following this research line, co-nanoencapsulation of sorafenib with other compounds has been proposed [72,73,74].

**Figure 3:**
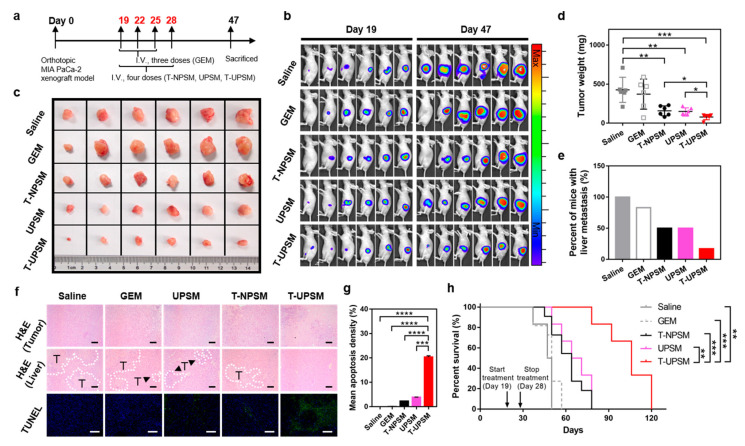
Antitumor efficacy of triptolide-loaded micelles in an orthotopic KRAS mutant MIA PaCa-2 cell-derived xenograft mouse model. (**a**) Scheme of treatment of different formulations. Mice were intravenously administrated with either gemcitabine (GEM) (50 mg/kg) for three doses or triptolide prodrug-loaded non-pH-sensitive micelles (T-NPSM) (0.3 mg/kg), ultra-pH-sensitive micelles (UPSM) (10 mg/kg), and triptolide prodrug-loaded UPSM (T-UPSM) (0.3 mg/kg) every other day for a total of four doses. (**b**) Bioluminescence images of anesthetized mice before (day 19) and after (day 47) treatments. Day 0 was designated as the day after injection of MIA PaCa-2-luc cells. (**c**) Ex vivo tumor pictures and (**d**) tumor weights after mice were randomly selected and sacrificed at the end of the experiment. Data are presented as the mean ± SD (*n* = 6). * *p* < 0.05, ** *p* < 0.01, *** *p* < 0.001, two-tailed *t* test. (**e**) Percentages of liver metastasis in different treatment groups. (**f**) Representative images of ex vivo histological (H&E and TUNEL staining) analyses of tumor sections. The areas surrounded by a white dotted line (marked with T) represent metastatic tumors. Scale bar: 50 μm. (**g**) Quantity analysis of apoptosis density in TUNEL staining by ImagePro Plus (*n* = 6 random fields). Data are presented as the mean ± SD (*** *p* < 0.001, **** *p* < 0.0001, two-tailed *t* test). (**h**) Kaplan–Meier survival curve of orthotopic xenograft models (*n* = 6 for saline, UPSM, and T-UPSM; *n* = 11 for GEM and T-NPSM) after treatment with the above formulations. Statistical significance for survival analysis was calculated using the log-rank test: ** *p* < 0.01, *** *p* < 0.001. Reprinted from [77] with permission of American Chemical Society.

To study the therapeutic potential of doxorubicin (DOX) to treat KRAS-mutated cells, DOX was loaded into gold nanoparticles (AuNP) modified with polyethylene glycol (PEG) and polyethylenimine (PEI) (AuPPPy-DOX) [75]. This system was able to reduce the viability of DLD-1 and HCT-116 cell lines (both KRAS-mutated) and to promote cell cycle arrest in the G2 phase. Moreover, in a DLD-1 subcutaneously implanted mice model, a statistically significant inhibition of the tumor growth (superior to 65%) was observed in animals treated with AuPPPy-DOX compared to the 30% inhibition observed with animals treated with free DOX. In addition, no signs of toxicity were noticed during the experimental period. In another study, camptothecin was encapsulated into nanoparticles composed of hydroxyethyl starch conjugated with lauric acid and L-leucine [76]. The system preferentially released the drug at the pH of the tumor microenvironment and significantly inhibited the expression of KRAS in an in vivo transgenic zebrafish model of hepatic cancer. The development of systems that respond to the pH of the microenvironment was also explored by Kong, C, et al. (2019) to deliver triptolide to KRAS-mutated pancreatic cancer cells [77]. Poly-(ethylene glycol)-block-poly(dipropylaminoethyl methacrylate) block copolymer (PEG-b-PDPA) micelles are spherical at pH 7.4 and suffer protonation and dissociation into unimers, with the consequent release of the drug, at pH 5.0. Thus, they promote a preferential release of the drug at the tumoral site. In an orthotopic KRAS mutant MIA PaCa-2 cell-derived xenograft mouse model, the pH-responsive micelles promoted a higher inhibition of the tumor growth and induced the apoptosis of tumor cells to a higher extent than the non-responsive system. More importantly, the treatment suppressed the formation of liver metastasis and prolonged the survival of animals (Figure 3).

Gemcitabine is a nucleoside analog and a first-line therapy for pancreatic cancer. However, its therapeutic efficacy is limited due to poor penetration into tumors and the development of resistance [78,79], which makes it a good candidate for nanodelivery. For that, Das, M, et al. (2020) developed liposomes with calcium phosphate for delivery of gemcitabine to improve the treatment of KRAS-mutated pancreatic cancer [80]. The formulation allowed the bypass of the hallmarks of gemcitabine chemoresistance and led to robust tumor regression in an aggressive and clinically relevant pancreatic ductal adenocarcinoma (PDAC) model.

**Figure 4:**
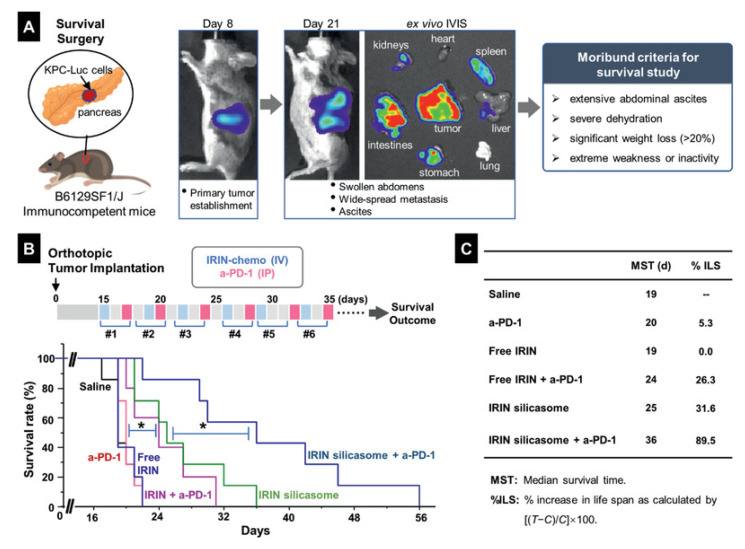
Animal survival study in an orthotopic KRAS-induced pancreatic cancer (KPC) model, treated with irinotecan (IRIN) silicasome plus anti-PD-1 antibody. (**A**) Explanation of the KPC model, including orthotopic implant in the pancreas and technical development of the primary tumor and metastases that can be followed by IVIS imaging. Animals were sacrificed according to the established moribund criteria. (**B**) Details of the survival experiment in tumor-bearing mice (*n* = 5–7), which were treated with free IRIN or the silicasome at an IRIN dose equivalent of 40 mg kg^−1^ IV every 3 or 4 days, with or without IP administration of 100 µg anti-PD-1 antibody, for a total of six administrations. Please notice that the antibody was administered two days after IRIN injection. Saline and anti-PD-1 alone were used as controls. Kaplan–Meier plots were used to display the survival rate of the different animal groups (* *p* < 0.05, log-rank test). (**C**) Summary of the median survival time (MST) and percentage of increase in life span (%ILS) for each group. Reprinted from [81] with permission of John Wiley and Sons under Creative Commons Attribution License (CC BY 4.0).

Combined chemo- and immunotherapy using nanostructured irinotecan and an anti-PD-1 compound to treat PDAC was also assessed [81]. The encapsulation of irinotecan into lipid bilayer-coated mesoporous silica nanoparticles (silicasome) led to an improvement of the delivery and therapeutic efficacy both in vitro and in vivo in KRAS-induced pancreatic cancer cell models. Irinotecan promotes lysosomal alkalization, leading to the inhibition of autophagy and immunogenic cell death induction. A synergic effect in the immune response was observed with a concomitant administration of an anti-PD-1 compound. In vivo, the combined therapy significantly improved the survival of animals compared to the monotherapy of both compounds (Figure 4). Similar results were obtained with the same system but substituting irinotecan with platinum-based compounds [82].

### 4.2. Biotechnological/Biopharmaceutical Therapy

Biological products, also referred to as “biologics” or “biopharmaceuticals”, contain an active substance derived from or extracted from a biological system (living organism), including animal and plant cells, bacteria, yeast, or viruses. However, nowadays it is more common to produce them using recombinant DNA technologies [83]. The first approved biopharmaceutical obtained through biotechnology-based techniques was the recombinant human insulin in 1982, and at the moment there are more than 300 distinct active biopharmaceutical ingredients already approved. The field of biopharmaceutics includes monoclonal antibodies, vaccines, hormones, interferons, blood factors, hematopoietic growth factors, genetic material, and thrombolytic agents [83,84]. Amongst the different types of biopharmaceuticals, monoclonal antibodies correspond to approximately 50% of biopharmaceuticals under development, under approval, and approved for clinical practice [84,85].

Given the higher specificity of this type of therapy and the increasing need for more personalized medicines, the use of these products in clinical practice has grown exponentially, with a consequent decrease in the use of conventional drugs [84]. However, due to their high sensitivity, lack of stability, and difficulties in crossing biological membranes, it is of major importance to find the perfect solution to protect and deliver biologic products in their active form into the cell of interest. In this sense, nanotechnology-based formulations are a good alternative to improve the stability and permeability of biological compounds. Moreover, nanoparticles allow the efficient internalization of these products in the target cells, becoming a great tool in the development of new biopharmaceutical-based formulations [86].

#### 4.2.1. Peptide/Protein-Based Compounds

**Figure 5:**
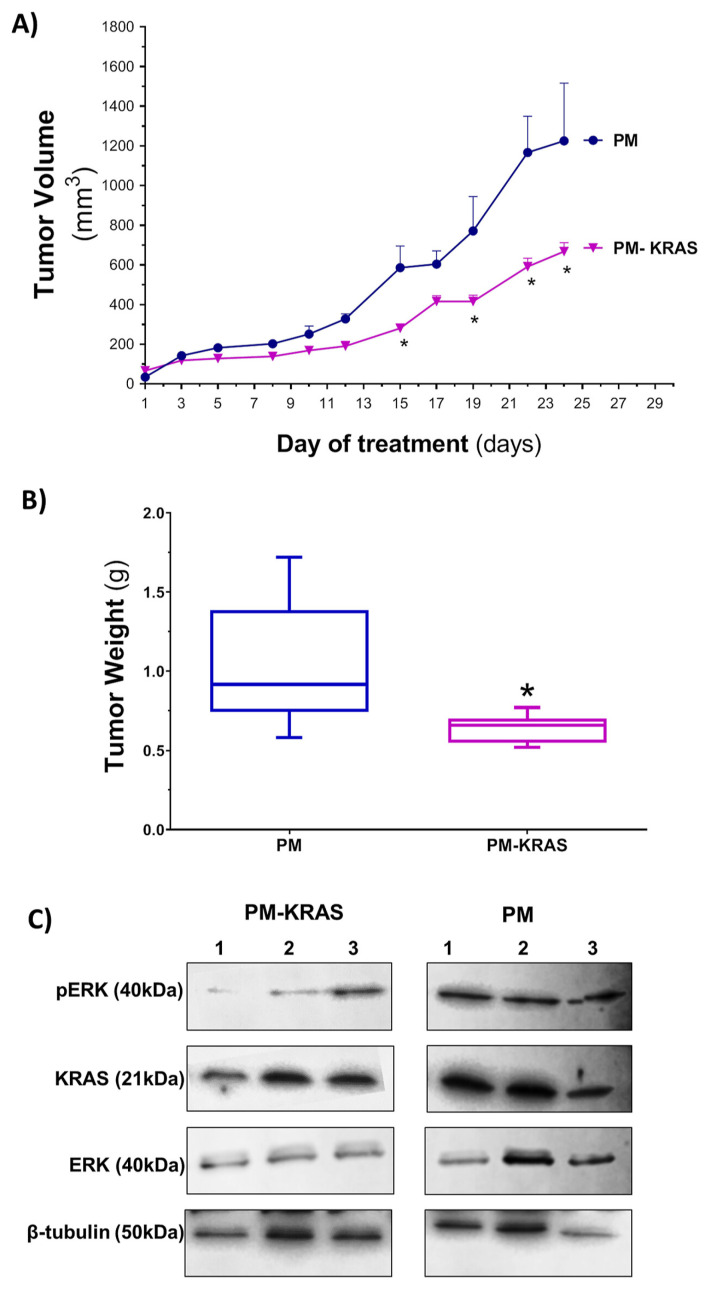
Therapeutic efficacy of polymeric micelles loaded with anti-KRAS antibody (PM-KRAS) in subcutaneous HCT116 tumors. (**A**) Tumor growth in animals treated intravenously with 300 mg/kg empty polymeric micelles (PMs) or PMs encapsulating anti-KRAS antibody (PM-KRAS) (300 μg/kg). Tumor volume was measured on the treatment days. (**B**) Ex vivo tumor weight at the end point of the treatment. (**C**) Protein levels in selected tumors of PM- and PM-KRAS-treated mice. Results are presented as mean ± SEM, *n* = 10, * denotes significant differences (*p* < 0.05) in tumor volume and tumor weight between both groups. Reprinted from [87] with permission of American Chemical Society.

Over the last few years, different antibodies, peptides, nanobodies, and affimers have been designed to selectively bind to KRAS and its mutated forms in order to block its biological activity in a specific manner [87,88,89,90,91,92,93]. In order to improve their internalization into the cells, different approaches have been used. An example of that is the recent work of Rafael, D, et al. (2023) in which Pluronic F127-based polymeric micelles were used for the encapsulation of anti-KRAS antibodies (PM-KRAS) [87]. They demonstrated high efficacy in vitro in terms of proliferation and colony formation inhibition for colon and pancreatic cancer cells. These results demonstrate not only a strong downregulation of the RAS/MAPK pathway, but also a stemness phenotype in the cell, as demonstrated by the gene expression levels. Moreover, in an in vivo colon cancer model, they demonstrated a significant reduction in tumor growth for the animals treated with the micelles encapsulating the anti-KRAS antibody in comparison with the animals receiving the empty micelles (Figure 5) [87]. This work was a clear example of how the antibodies have great potential not only for extracellular targets but also for intracellular targets, especially the ones considered undruggable or untargetable using other therapeutic options. Other groups are also pursuing the intracellular delivery of anti-KRAS antibodies. For example, Libera Bio patented a nanocapsule system based on an oil core and a hydrophilic polymeric shell (MPN technology) to deliver an anti-KRAS antibody targeting the G12V mutation [94]. In vivo, the system promotes a reduction in tumor volume [94,95].

Peptides against KRAS may also benefit from the use of nanoDDSs. Sakamoto, K, et al. (2023) nanoformulated the K-Ras(G12D)-inhibitory bicyclic peptide KS-58 into micelles that demonstrated antitumor activity against colon and pancreatic tumors [88]. Their results show that the KS-58 nanoparticles accumulated into tumors and suppressed the growth of CT26 and PANC-1 tumors in vivo.

#### 4.2.2. Gene Therapy

Since KRAS presents a variety of already known mutations, in order to promote a mutation-specific treatment, many research groups have been putting efforts into developing new therapeutic solutions based on gene therapy that in most cases require nanoformulation. This, together with the small amount of small molecule/chemical inhibitors discovered so far, explains why most nanoparticulated anti-KRAS therapies are non-viral vectors developed to be used alone or in combination with chemotherapy [96,97,98,99]. The use of nanotechnology to create non-viral vectors for gene therapy allows the substitution of viral vectors and their associated toxicity. The development of non-viral vectors for KRAS-mutated cancer treatment has exponentially grown in the last decade [100,101,102], with the aim of (i) suppressing gene expression at the mRNA level (RNA interference strategies), (ii) artificially increasing gene expression, or (iii) correcting defective genes (gene modification). The advantage over other treatment approaches is that it is highly specific and suitable for developing mutation-specific treatment strategies.

##### Gene Silencing

**Figure 6:**
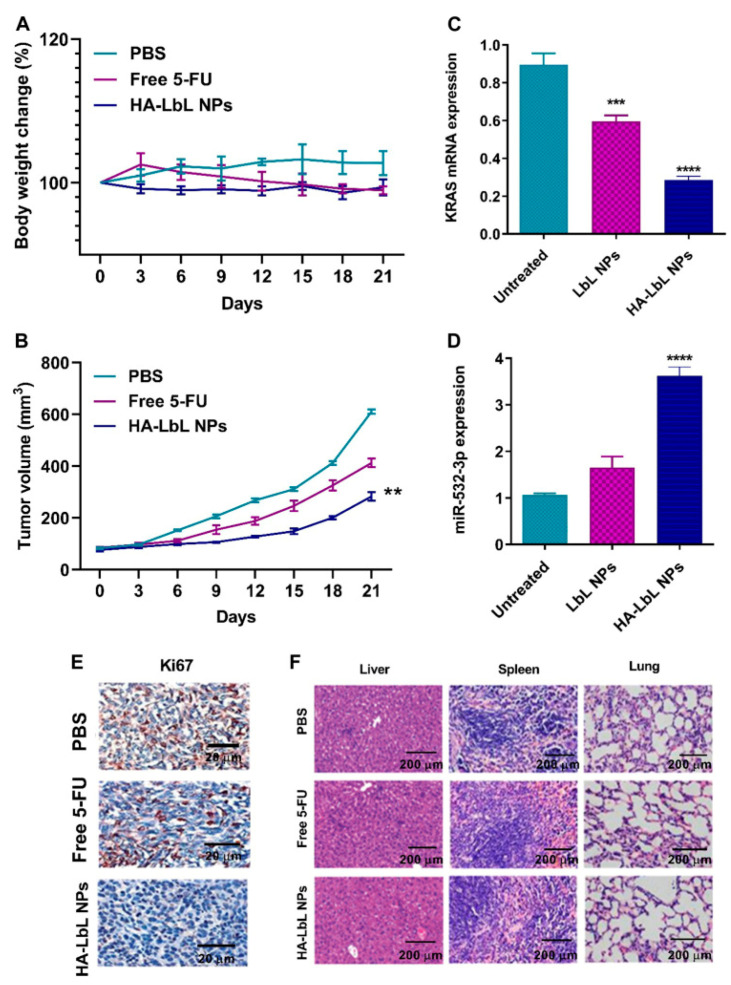
In vivo antitumor evaluation of LbL formulation in tumor-bearing mice after intravenous injection with PBS, free 5-FU, and HA-LbL NPs (*n* = 5/each group). (**A**) Body weight curve. (**B**) Tumor volume curve. (**C**) Relative expression of KRAS. (**D**) Relative expression of miR-532-3p. (**E**) Immunostaining of Ki67. (**F**) H&E staining of liver, spleen, and lung. ** *p* < 0.01, *** *p* < 0.01, **** *p* < 0.01. Abbreviations: HA: hyaluronic acid, LbL NP: layer-by-layer nanoparticle, 5-FU: 5-Fluorouracil), PBS: phosphate buffer solution. Reprinted from [99] with permission of Frontiers Media under Creative Commons Attribution License (CC BY 4.0).

Several types of nanoparticles have been used as non-viral gene delivery vectors; the ones with a lipidic nature are the most commonly used both in clinical research and clinical practice. Regarding nanomedicines for targeting KRAS-mutated cancers, a lipid nanoparticle comprising an siRNA therapeutic against glutathione S-transferase P (NBF-006) was able to promote a tumor regression and a prolongation in the survival rate of animals in a surgically implanted orthotopic NSCLC tumor model [103]. This system was recently enrolled in a Phase I clinical trial (NCT03819387) with NSCLC, CRC, and pancreatic cancer patients. Another example is the work of Shahidi, M, et al. (2022) on the design of a liposome coated by cationic chitosan (CS) using a controlled layer-by-layer (LbL) process to deliver simultaneous siKRAS, miRNA, and 5-Fluorouracil (5-FU) into CRC cells [99]. MiR-532-3p acts as a sensitizer to 5-FU in CRC through its activating effects on p53 overcoming treatment resistance. In vitro, the LbL NPs were able to internalize and promote cytotoxicity, suppressing cancer cell migration and invasion. In vivo, there was reduced tumor growth in treated SW480-tumor-bearing mice models. The strategy exhibited significant tumor inhibition efficiency without remarkable changes in body weight and organ toxicity (Figure 6). Compared to the free 5-FU formulation used alone, the co-delivery of the 5-FU and miR-532-3p/si-KRAS greatly improved antitumor efficacy. This new nanoDDS is expected to be a good system with great potential for the synergic treatment of CRC.

**Figure 7:**
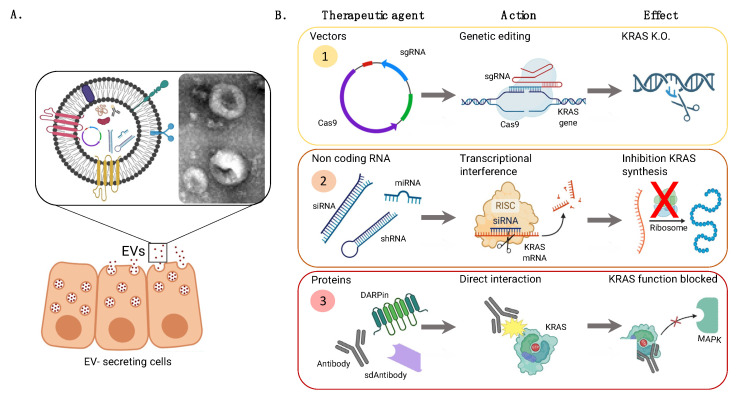
EV potential uses for enabling KRAS targeting. (**A**). Schematic representation of EV secretion from mammalian cells accompanied by a detailed TEM image of EVs after isolation by size exclusion chromatography. (**B**). Pathways to target KRAS using EVs as nanotechnology drug delivery systems.

Extracellular vesicles (EVs), which present some structural similarities to liposomes, represent a promising alternative as a gene delivery platform. They are relatively inert, non-immunogenic, biodegradable, and biocompatible [104] and can be used for targeting KRAS by different strategies (Figure 7). Mendt, M, et al. (2018) proposed KRAS G12D inhibition by the administration of EVs isolated from mesenchymal stromal cells and loaded with a specific siRNA [105]. This strategy has been proven efficacious in reducing tumor burden in pancreatic cancer models and is fully scalable [106]. Of note, this system is currently undergoing Phase I clinical trials (NCT03608631) [45].

Other types of nanoparticles developed for the delivery of siRNA towards KRAS-mutated cells consist of macromolecular assemblies of biological origin such as albumin. However, only in vitro data are available, and further studies would be necessary to assess their potential [107]. Closely related molecules such as peptides have also shown the ability to generate nanoDDSs for siRNA delivery. In this regard, Strand, MS, et al. (2019) were able to generate NPs out of a p5RHH cationic peptide, engineered from natural melittin structure. The resulting NPs were able to load a specific siRNA against KRAS, showing significant efficacy in in vitro and in vivo PDAC models [108].

Another promising strategy of gene therapy against KRAS-mutated cancer consists in the use of microRNAs (miRs). For example, miR-143 has been shown to act as a tumor suppressor in NSCLC, cervical cancer, prostate cancer, ovarian cancer, colon cancer, and leukemia, being able to silence not only KRAS but also RAS-effector signal genes Erk and Akt [109]. In this sense, Yoshikawa, Y, et al. (2019) developed a novel chemically modified miR-143 (miR-143#12) that exhibited a marked antitumor activity upon either systemic or intravesical administration with a polyionic copolymer (PIC) as the carrier in KRAS-driven bladder cancer [110]. PIC micelles can be prepared through the spontaneous assembly of cationic block copolymers with oppositely charged miRNA. Their core–shell architectures offer a delivery platform for vulnerable miRNA, improving their biological activities for medicinal applications such as tumor-targeted therapy. They also used the same nanocarrier to treat renal cell carcinoma (RCC) in Caki-1 cell-xenografted mice and found that PIC could protect synthesized miR-143s in the blood and the treatment exhibited a marked antitumor effect, as observed in in vitro experiments [111].

##### Gene Editing

CRISPR (clustered, regularly interspaced, short palindromic repeats)-associated 9 (Cas9)-based technology has emerged as a precise therapeutic tool. However, its clinical translation remains a challenge due to difficulties in the successful and safe intracellular delivery of the system, especially in the form of ribonucleoprotein (RNP) [112,113]. The delivery of RNP circumvents the processes of transcription and translation, generating a rapid genome-editing effect both in vitro and in vivo. Furthermore, Cas9 RNP is free of insertional mutagenesis and shows low off-target effects, making it an appealing delivery format.

Wan, T, et al. (2021) designed a hyaluronic acid (HA)-decorated phenylboronic dendrimer (HAPD) to deliver Cas9 RNP to target both concurrent adenomatous polyposis coli (APC) and KRAS mutations. The systemic administration of duplex Cas9 RNP by HAPD was able to inhibit tumor growth on xenografted and orthotopic CRC mouse models and to prevent CRC-induced liver and lung metastasis [114]. Previously, they had already designed a disulfide-bridged biguanidyl adamantine (Ad-SS-GD) with a β-cyclodextrin-conjugated low-molecular-weight polyethyleneimime (CP) nanocomplex with high efficiency for in vitro cytosolic delivery of RNP, and it was able to inhibit tumor growth and metastasis in the tumor-bearing CRC mouse models [112].

EVs have also been used to deliver CRISPR/Cas9 vector (Figure 7), coding for Cas9 plasmid and sgRNA specific for the KRAS G12D mutation, to disrupt KRAS activity at the gene level in in vitro and in vivo pancreatic cancer models [115].

**Table 2:** Examples of nanoparticles for anti-KRAS therapy in different stages of development.

Category	Vehicle	Cargo	Application	Development Stage	Reference
Chemotherapy	Amphiphilic hydroxyethyl starch-conjugated lauric acid and L-leucine NP	Camptothecin	Hepatic cancer	In vivo	[76]
	PLGA NP	Afatinib	NSCLC	In vitro	[61]
	Gold NP	Doxorubicin	CRC	In vivo	[75]
	Liposomes (DOPA, DOTAP, Chol, DSPE-PEG) with calcium phosphate	Gemcitabine	PDAC	In vivo	[80]
	Silicasomes (DSPC/Chol/DSPE-PEG liposomes with mesoporous silica)	Irinotecan and an anti-PD-1 compound	PDAC	In vivo	[81]
	Silicasomes (DSPC/Chol/DSPE-PEG liposomes with mesoporous silica)	Platinum-based compounds and an anti-PD-1 compound	PDAC	In vivo	[82]
	PLGA-coated gold NP	5-Fluorouracil	Lung cancer	In vitro	[116]
	PEG-b-PDPA micelles	Triptolide	PDAC	In vivo	[77]
	Cetuximab-conjugated PEG-PLGA NP	Camptothecin	Pancreatic cancer	In vivo	[117]
	Albumin NP	β-lapachone	PDAC	In vivo	[60]
	PpIX-C6-PEG8-KKKKKKSKTKC-OMe peptidic micelles	Protoporphyrin IX	Breast cancer	In vivo	[118]
	Avidin–nucleic acid nanoassemblies	Doxorubicin	Breast cancer	In vivo	[119]
Peptide/protein-based therapy	Pluronic-based micelles	Anti-KRAS antibody	CRC and pancreatic cancer	In vivo	[87]
	Cremophor EL-based micelles	Bicyclic peptide KS-58	CRC and pancreatic cancer	In vivo	[88]
	MPN technology nanocapsules	Anti-KRAS antibody	PDAC	In vivo	[94]
Gene therapy	Albumin NP	siKRAS	Lung cancer therapy	In vitro	[107,114]
	p5RHH NP	siKRAS	Pancreatic Cancer	In vivo	[108]
	EVs	siKRAS	Pancreatic Cancer	Phase I	[45]
	Lipid NP	siKRAS and gemcitabine	Pancreatic cancer	In vivo	[97,107]
	PEI-modified hydroxyapatite NP	siKRAS	Pancreatic cancer	In vitro	[108,120]
	Cationic poly (cyclohexene carbonate) NP	siKRAS	Pancreatic cancer	In vitro	[45,121]
	Antibody-cationized gelatin NP	siKRAS	NSCLC	In vitro	[97,122]
	HA layer-by-layer liposomes	siKRAS miR-532-3p 5-Fluorouracil (5-FU)	CRC	In vivo	[99,120]
	Polyionic copolymer nanocarrier	miR-143#12	Bladder cancer and RCC	In vivo	[110]
	PAMAM dendrimer	miRNA Mimic let-7b chloroquine	NSCLC	In vitro	[98]
	HA-decorated HAPD	Cas9 RNP sgRNAs targeting mutant APC and KRAS	CRC	In vivo	[114]
	Disulfide-bridged biguanidyl adamantine with β-cyclodextrin-conjugated low-molecular-weight polyethyleneimime nanocomplex	Cas9 RNP sgRNAs targeting mutant KRAS	CRC	In vivo	[112]
	EVs	CRISPR/Cas9 vector (LentiCRISPR V2 and pSpCas9(BB)-2A-GFP (PX458))	Pancreatic cancer	In vivo	[115]
	Thiol-modified glycol chitosan NP	siKRAS and GDC-0941	Ovarian cancer	In vivo	[102]
	Lipid NP	siGSTP	NSCLC, CRC, and pancreatic cancer	Phase I clinical trial	[103]

Abbreviations: Chol—cholesterol, CRC—colorectal cancer, DOPA—dioleoylphosphatidic acid, DOTAP—1,2-dioleoyl-3-trimethylammonium-propane, DSPC—1,2-distearoyl-sn-glycero-3-phosphocholine, DSPE-PEG—N-(methylpolyoxyethylene oxycarbonyl)-1,2-distearoyl-sn-glycero-3-phosphoethanolamine, HA—hyaluronic acid, HAPD—phenylboronic dendrimer, NSCLC—non-small cell lung cancer, NP—nanoparticle, PAMAM—polyamidoamine, PDAC—pancreatic ductal adenocarcinoma, PEG—polyethylene glycol, PEG-b-PDPA—poly-(ethylene glycol)-block-poly(dipropylaminoethyl methacrylate) block copolymer, PEI—polyethylenimine, PLGA—polylactic-co-glycolic acid, RCC—renal cell carcinoma, RNP—ribonucleoprotein, sgRNA—single-guide RNA, siGSTP—siRNA against glutathione S-transferase P.

## 5. Nanomedicine Challenges

Nanomedicine offers promising opportunities for improving KRAS-mutated cancer therapy, but there are still several limitations and challenges that must be overcome related to toxicity, targeted delivery, stability, manufacturing and scale-up, and regulatory approval [49,123,124,125]. One of the main concerns with nanomedicine is the potential for toxicity, especially when it comes to long-term exposure. While nanomaterials can be engineered to be biocompatible, there is still a risk that they may cause accumulation and damage to healthy cells or tissues [124]. However, since nanoparticles for treating KRAS-mutated cells are expected to be used in short-time therapies, the efficacy/safety ratio is still positive. Another challenge is ensuring that the nanoparticles reach the target site at therapeutic doses. Many types of cancer therapy require the drug to be delivered specifically to the tumor cells, while avoiding healthy cells. However, the body’s natural defenses, such as the immune system and the blood–brain barrier, can prevent the nanoparticles from reaching their intended destination [123]. Nevertheless, although not all nanoparticles reach the site of interest, they modify the biodistribution of a drug, improving its therapeutic index. Stability, both under storage and in the presence of biological fluids, could be a limitation for some nanoparticles. This can cause aggregation, opsonization, or degradation that can affect their effectiveness or safety profile [49]. For this reason, during the development phase, deeply studying the stability of the systems under different conditions to avoid problems in the translation to in vivo conditions is of the utmost importance.

One of the main concerns regarding nanomedicine is related to the manufacturing processes used and its scale-up to the industrial level [125]. Some manufacturing methods can be complex and expensive and may not be able to fulfill the demand for large-scale clinical trials or commercialization. In addition, at the regulatory level, despite the efforts made in the last few years and the presence of nanomedicines in the market, there is still a lack of standardization in the characterization and evaluation of nanomedicines that hinders regulatory approval [125]. Additionally, the mentioned concerns around the safety and efficacy of these therapies can further slow the approval process.

## 6. Conclusions and Future Perspectives

The therapeutic arsenal under development to inactivate the different KRAS mutations is wide, including small drugs, antibodies, and gene therapy. However, the majority of these therapies may require nanoformulation to improve their pharmacokinetic properties, tumor specificity, and biological efficacy. Although fairly explored, the use of nanomedicine to improve the therapies targeting mutated KRAS is a field expected to increase in the next years. Likewise, the plethora of nanoparticulate systems that can be used is very broad, including not only lipidic, polymeric, and inorganic synthetic nanoparticles, but also naturally obtained EVs and biological-based particles. Presently, there is not a clear candidate, in terms of therapeutic cargo or in terms of delivery system, for each clinical application. In this sense, the endless opportunities that nanomedicines create to find a newer, efficient, and specific treatment bring the hope that, in the near future, more therapeutic options will be available against KRAS-mutated cancers, still considered an important clinical challenge.

## Figures and Tables

**Table 1:** Examples of nanoparticles used in the development of anti-KRAS-based therapies, their advantages, disadvantages, and diagram representation.

Type	Advantages	Disadvantages	Diagram
Liposomes	Biocompatibility. Biodegradability. Co-loading of drugs with different polarities.	Difficult and high production costs. Storage stability. Leakage of drugs.	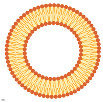
Solid Lipid Nanoparticles	Biocompatibility. Biodegradability. Simultaneous loading of drugs with different polarities.	Low loading of hydrophilic drugs. Long-term stability (crystallization).	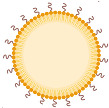
Polymeric Nanoparticles	Controlled release properties. High stability.	Difficulties in scale-up. Potentially more toxic. Low loading capacity.	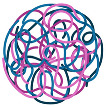
Polymeric Micelles	Co-loading of drugs with different polarities. Easy and cheap preparation.	Loading limitations to some drugs.	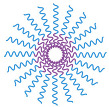
Dendrimers	High drug loading. Versatility of surface functionalization.	High toxicity. Hemolytic properties. Non-biodegradability.	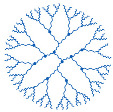
Extracellular Vesicles	Biocompatibility. Biodegradability. Stability. Versatility of drug loading. Versatility of surface functionalization.	Difficult and high production costs. Heterogeneity in production. Difficulties in scale-up.	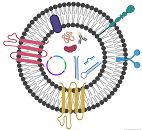
Protein and Peptide Nanoparticles	Biocompatibility. Biodegradability. Versatility of functionalization.	High production cost. Stability.	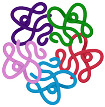
Inorganic Nanoparticles	Versatility of surface functionalization. Stimuli-responsiveness.	Long-term toxicity. Stability. Non-biodegradability.	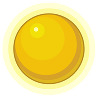
Nucleic acid Nanoparticles	Biocompatibility. Biodegradability. Versatility of functionalization.	Stability. High production cost.	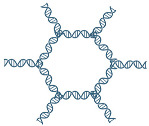

## Data Availability

Not applicable.

## References

[1] Hobbs G.A., Der C.J., Rossman K.L. (2016). RAS isoforms and mutations in cancer at a glance. J. Cell Sci..

[2] Cox A.D., Fesik S.W., Kimmelman A.C., Luo J., Der C.J. (2014). Drugging the undruggable RAS: Mission possible?. Nat. Rev. Drug Discov..

[3] Braicu C., Buse M., Busuioc C., Drula R., Gulei D., Raduly L., Rusu A., Irimie A., Atanasov A.G., Slaby O. (2019). A Comprehensive Review on MAPK: A Promising Therapeutic Target in Cancer. Cancers.

[4] Zhang W., Liu H.T. (2002). MAPK signal pathways in the regulation of cell proliferation in mammalian cells. Cell Res..

[5] Sun Y., Liu W.Z., Liu T., Feng X., Yang N., Zhou H.F. (2015). Signaling pathway of MAPK/ERK in cell proliferation, differentiation, migration, senescence and apoptosis. J. Recept. Signal Transduct. Res..

[6] Rodriguez-Viciana P., Warne P.H., Dhand R., Vanhaesebroeck B., Gout I., Fry M.J., Waterfield M.D., Downward J. (1994). Phosphatidylinositol-3-OH kinase as a direct target of Ras. Nature.

[7] Pacold M.E., Suire S., Perisic O., Lara-Gonzalez S., Davis C.T., Walker E.H., Hawkins P.T., Stephens L., Eccleston J.F., Williams R.L. (2000). Crystal structure and functional analysis of Ras binding to its effector phosphoinositide 3-kinase gamma. Cell.

[8] Hofer F., Fields S., Schneider C., Martin G.S. (1994). Activated Ras interacts with the Ral guanine nucleotide dissociation stimulator. Proc. Natl. Acad. Sci. USA.

[9] Neel N.F., Martin T.D., Stratford J.K., Zand T.P., Reiner D.J., Der C.J. (2011). The RalGEF-Ral Effector Signaling Network: The Road Less Traveled for Anti-Ras Drug Discovery. Genes Cancer.

[10] Jancík S., Drábek J., Radzioch D., Hajdúch M. (2010). Clinical relevance of KRAS in human cancers. J. Biomed. Biotechnol..

[11] Fang B. (2016). RAS signaling and anti-RAS therapy: Lessons learned from genetically engineered mouse models, human cancer cells, and patient-related studies. Acta Biochim. Biophys. Sin..

[12] Muñoz-Maldonado C., Zimmer Y., Medová M. (2019). A Comparative Analysis of Individual RAS Mutations in Cancer Biology. Front Oncol.

[13] Khan A.Q., Kuttikrishnan S., Siveen K.S., Prabhu K.S., Shanmugakonar M., Al-Naemi H.A., Haris M., Dermime S., Uddin S. (2019). RAS-mediated oncogenic signaling pathways in human malignancies. Semin. Cancer Biol..

[14] Karnoub A.E., Weinberg R.A. (2008). Ras oncogenes: Split personalities. Nat. Rev. Mol. Cell Biol..

[15] Prior I.A., Lewis P.D., Mattos C. (2012). A comprehensive survey of Ras mutations in cancer. Cancer Res..

[16] Meng M., Zhong K., Jiang T., Liu Z., Kwan H.Y., Su T. (2021). The current understanding on the impact of KRAS on colorectal cancer. Biomed. Pharmacother..

[17] Stolze B., Reinhart S., Bulllinger L., Fröhling S., Scholl C. (2015). Comparative analysis of KRAS codon 12, 13, 18, 61, and 117 mutations using human MCF10A isogenic cell lines. Sci. Rep..

[18] Huang L., Guo Z., Wang F., Fu L. (2021). KRAS mutation: From undruggable to druggable in cancer. Signal. Transduct. Target. Ther..

[19] Pantsar T. (2020). The current understanding of KRAS protein structure and dynamics. Comput. Struct. Biotechnol. J..

[20] Karimi N., Moghaddam S.J. (2023). KRAS-Mutant Lung Cancer: Targeting Molecular and Immunologic Pathways, Therapeutic Advantages and Restrictions. Cells.

[21] Tang D., Kroemer G., Kang R. (2021). Oncogenic KRAS blockade therapy: Renewed enthusiasm and persistent challenges. Mol. Cancer.

[22] Ghimessy A., Radeczky P., Laszlo V., Hegedus B., Renyi-Vamos F., Fillinger J., Klepetko W., Lang C., Dome B., Megyesfalvi Z. (2020). Current therapy of KRAS-mutant lung cancer. Cancer Metastasis Rev..

[23] Uprety D., Adjei A.A. (2020). KRAS: From undruggable to a druggable Cancer Target. Cancer Treat. Rev..

[24] Nakajima E., Drezner N., Li X., Mishra-Kalyani P., Liu Y., Zhao H., Bi Y., Liu J., Rahman A., Wearne E. (2022). FDA Approval Summary: Sotorasib for KRAS G12C-Mutated Metastatic NSCLC. Clin. Cancer Res. Off. J. Am. Assoc. Cancer Res..

[25] Tanaka N., Lin J., Li C., Ryan M., Zhang J., Kiedrowski L., Michel A., Syed M., Fella K., Sakhi M. (2021). Clinical Acquired Resistance to KRASG12C Inhibition through a Novel KRAS Switch-II Pocket Mutation and Polyclonal Alterations Converging on RAS-MAPK Reactivation. Cancer Discov..

[26] Lee A. (2022). Sotorasib: A Review in KRAS G12C Mutation-Positive Non-small Cell Lung Cancer. Target. Oncol..

[27] Jänne P.A., Riely G.J., Gadgeel S.M., Heist R.S., Ou S.I., Pacheco J.M., Johnson M.L., Sabari J.K., Leventakos K., Yau E. (2022). Adagrasib in Non-Small-Cell Lung Cancer Harboring a KRAS G12C Mutation. N. Engl. J. Med..

[28] Dhillon S. (2023). Adagrasib: First Approval. Drugs.

[29] Khan H.Y., Nagasaka M., Li Y., Aboukameel A., Uddin M.H., Sexton R., Bannoura S., Mzannar Y., Al-Hallak M.N., Kim S. (2022). Inhibitor of the Nuclear Transport Protein XPO1 Enhances the Anticancer Efficacy of KRAS G12C Inhibitors in Preclinical Models of KRAS G12C-Mutant Cancers. Cancer Res. Commun..

[30] Patricelli M.P., Janes M.R., Li L.S., Hansen R., Peters U., Kessler L.V., Chen Y., Kucharski J.M., Feng J., Ely T. (2016). Selective Inhibition of Oncogenic KRAS Output with Small Molecules Targeting the Inactive State. Cancer Discov..

[31] Janes M.R., Zhang J., Li L.S., Hansen R., Peters U., Guo X., Chen Y., Babbar A., Firdaus S.J., Darjania L. (2018). Targeting KRAS Mutant Cancers with a Covalent G12C-Specific Inhibitor. Cell.

[32] Li L., Zhao H., Liao H., Chen J., Liu J. (2021). Discovery of novel quinazoline-based covalent inhibitors of KRAS G12C with various cysteine-targeting warheads as potential anticancer agents. Bioorg. Chem..

[33] Li L., Zhao H., Peng X., Liu J., Mai R., Chen J., Lin L., Chen T., Yan J., Shi J. (2022). Discovery of novel Quinazoline-based KRAS G12C inhibitors as potential anticancer agents. Bioorg. Med. Chem..

[34] Zhao H., Li L., Liu J., Mai R., Chen J. (2022). Discovery of ARS-1620 analogs as KRas G12C inhibitors with high in vivo antitumor activity. Bioorg. Chem..

[35] Li W., Chen W., Wang J., Zhao G., Chen L., Wan Y., Luo Q., Huang H., Yang Y., Chen D. (2022). A PDX model combined with CD-DST assay to evaluate the antitumor properties of KRpep-2d and oxaliplatin in KRAS (G12D) mutant colorectal cancer. Heliyon.

[36] Sakamoto K., Lin B., Nunomura K., Izawa T., Nakagawa S. (2022). The K-Ras(G12D)-inhibitory peptide KS-58 suppresses growth of murine CT26 colorectal cancer cell-derived tumors. Sci. Rep..

[37] Sakamoto K., Masutani T., Hirokawa T. (2020). Generation of KS-58 as the first K-Ras(G12D)-inhibitory peptide presenting anti-cancer activity in vivo. Sci. Rep..

[38] Teng K.W., Tsai S.T., Hattori T., Fedele C., Koide A., Yang C., Hou X., Zhang Y., Neel B.G., O’Bryan J.P. (2021). Selective and noncovalent targeting of RAS mutants for inhibition and degradation. Nat. Commun..

[39] Guo C., Banerji U. (2021). Searching for treatments for non-G12C-KRAS mutant cancers. Br. J. Cancer.

[40] Zhou C., Fan Z., Zhou Z., Li Y., Cui R., Liu C., Zhou G., Diao X., Jiang H., Zheng M. (2022). Discovery of the First-in-Class Agonist-Based SOS1 PROTACs Effective in Human Cancer Cells Harboring Various KRAS Mutations. J. Med. Chem..

[41] Salgia R., Pharaon R., Mambetsariev I., Nam A., Sattler M. (2021). The improbable targeted therapy: KRAS as an emerging target in non-small cell lung cancer (NSCLC). Cell. Rep. Med..

[42] Ross S.J., Revenko A.S., Hanson L.L., Ellston R., Staniszewska A., Whalley N., Pandey S.K., Revill M., Rooney C., Buckett L.K. (2017). Targeting KRAS-dependent tumors with AZD4785, a high-affinity therapeutic antisense oligonucleotide inhibitor of KRAS. Sci. Transl. Med..

[43] Gort E., Johnson M.L., Hwang J.J., Pant S., Dünzinger U., Riemann K., Kitzing T., Janne P.A. (2020). A phase I, open-label, dose-escalation trial of BI 1701963 as monotherapy and in combination with trametinib in patients with KRAS mutated advanced or metastatic solid tumors. J. Clin. Oncol..

[44] Chen K., Zhang Y., Qian L., Wang P. (2021). Emerging strategies to target RAS signaling in human cancer therapy. J. Hematol. Oncol..

[45] Surana R., LeBleu V.S., Lee J.J., Smaglo B.G., Zhao D., Lee M.S., Wolff R.A., Overman M.J., Mendt M.C., McAndrews K.M. Phase I study of mesenchymal stem cell (MSC)-derived exosomes with KRAS G12D siRNA in patients with metastatic pancreatic cancer harboring a KRAS G12D mutation. Proceedings of the ASCO Gastrointestinal Cancers Symposium.

[46] Wang J., Martin-Romano P., Cassier P., Johnson M., Haura E., Lenox L., Guo Y., Bandyopadhyay N., Russell M., Shearin E. (2022). Phase I Study of JNJ-74699157 in Patients with Advanced Solid Tumors Harboring the KRAS G12C Mutation. Oncologist.

[47] Blaquier J.B., Cardona A.F., Recondo G. (2021). Resistance to KRAS G12C Inhibitors in Non-Small Cell Lung Cancer. Front. Oncol..

[48] Liu J., Kang R., Tang D. (2022). The KRAS-G12C inhibitor: Activity and resistance. Cancer Gene Ther..

[49] Rasool M., Malik A., Waquar S., Arooj M., Zahid S., Asif M., Shaheen S., Hussain A., Ullah H., Gan S.H. (2022). New challenges in the use of nanomedicine in cancer therapy. Bioengineered.

[50] Germain M., Caputo F., Metcalfe S., Tosi G., Spring K., Åslund A.K.O., Pottier A., Schiffelers R., Ceccaldi A., Schmid R. (2020). Delivering the power of nanomedicine to patients today. J. Control. Release.

[51] Bhatia S.N., Chen X., Dobrovolskaia M.A., Lammers T. (2022). Cancer nanomedicine. Nat. Rev. Cancer.

[52] Wu J. (2021). The Enhanced Permeability and Retention (EPR) Effect: The Significance of the Concept and Methods to Enhance Its Application. J. Pers. Med..

[53] Waheed S., Li Z., Zhang F., Chiarini A., Armato U., Wu J. (2022). Engineering nano-drug biointerface to overcome biological barriers toward precision drug delivery. J. Nanobiotechnology.

[54] Pearce A.K., O’Reilly R.K. (2019). Insights into Active Targeting of Nanoparticles in Drug Delivery: Advances in Clinical Studies and Design Considerations for Cancer Nanomedicine. Bioconjug. Chem..

[55] Shi L., Zhang J., Zhao M., Tang S., Cheng X., Zhang W., Li W., Liu X., Peng H., Wang Q. (2021). Effects of polyethylene glycol on the surface of nanoparticles for targeted drug delivery. Nanoscale.

[56] Suk J.S., Xu Q., Kim N., Hanes J., Ensign L.M. (2016). PEGylation as a strategy for improving nanoparticle-based drug and gene delivery. Adv. Drug. Deliv. Rev..

[57] Shi J., Kantoff P.W., Wooster R., Farokhzad O.C. (2017). Cancer nanomedicine: Progress, challenges and opportunities. Nat. Rev. Cancer.

[58] Liu X., Ghosh D. (2019). Intracellular nanoparticle delivery by oncogenic KRAS-mediated macropinocytosis. Int. J. Nanomed..

[59] Li R., Ng T.S.C., Wang S.J., Prytyskach M., Rodell C.B., Mikula H., Kohler R.H., Garlin M.A., Lauffenburger D.A., Parangi S. (2021). Therapeutically reprogrammed nutrient signalling enhances nanoparticulate albumin bound drug uptake and efficacy in KRAS-mutant cancer. Nat. Nanotechnol..

[60] Dou L., Liu H., Wang K., Liu J., Liu L., Ye J., Wang R., Deng H., Qian F. (2022). Albumin binding revitalizes NQO1 bioactivatable drugs as novel therapeutics for pancreatic cancer. J. Control. Release.

[61] Elbatanony R.S., Parvathaneni V., Kulkarni N.S., Shukla S.K., Chauhan G., Kunda N.K., Gupta V. (2021). Afatinib-loaded inhalable PLGA nanoparticles for localized therapy of non-small cell lung cancer (NSCLC)-development and in-vitro efficacy. Drug Deliv. Transl. Res..

[62] Maiti A., Naqvi K., Kadia T.M., Borthakur G., Takahashi K., Bose P., Daver N.G., Patel A., Alvarado Y., Ohanian M. (2019). Phase II Trial of MEK Inhibitor Binimetinib (MEK162) in RAS-mutant Acute Myeloid Leukemia. Clin. Lymphoma Myeloma Leuk..

[63] Sorokin A.V., Kanikarla Marie P., Bitner L., Syed M., Woods M., Manyam G., Kwong L.N., Johnson B., Morris V.K., Jones P. (2022). Targeting RAS Mutant Colorectal Cancer with Dual Inhibition of MEK and CDK4/6. Cancer Res..

[64] Dummer R., Schadendorf D., Ascierto P.A., Arance A., Dutriaux C., Di Giacomo A.M., Rutkowski P., Del Vecchio M., Gutzmer R., Mandala M. (2017). Binimetinib versus dacarbazine in patients with advanced NRAS-mutant melanoma (NEMO): A multicentre, open-label, randomised, phase 3 trial. Lancet Oncol..

[65] Bikhezar F., de Kruijff R.M., van der Meer A.J.G.M., Torrelo Villa G., van der Pol S.M.A., Becerril Aragon G., Gasol Garcia A., Narayan R.S., de Vries H.E., Slotman B.J. (2020). Preclinical evaluation of binimetinib (MEK162) delivered via polymeric nanocarriers in combination with radiation and temozolomide in glioma. J. Neurooncol..

[66] Dingemans A.M., Mellema W.W., Groen H.J., van Wijk A., Burgers S.A., Kunst P.W., Thunnissen E., Heideman D.A., Smit E.F. (2013). A phase II study of sorafenib in patients with platinum-pretreated, advanced (Stage IIIb or IV) non-small cell lung cancer with a KRAS mutation. Clin. Cancer Res..

[67] Nogova L., Mattonet C., Scheffler M., Taubert M., Gardizi M., Sos M.L., Michels S., Fischer R.N., Limburg M., Abdulla D.S.Y. (2020). Sorafenib and everolimus in patients with advanced solid tumors and KRAS-mutated NSCLC: A phase I trial with early pharmacodynamic FDG-PET assessment. Cancer Med..

[68] Kong F.H., Ye Q.F., Miao X.Y., Liu X., Huang S.Q., Xiong L., Wen Y., Zhang Z.J. (2021). Current status of sorafenib nanoparticle delivery systems in the treatment of hepatocellular carcinoma. Theranostics.

[69] Lai H., Zhong L., Huang Y., Zhao Y., Qian Z. (2021). Progress in Application of Nanotechnology in Sorafenib. J. Biomed. Nanotechnol..

[70] Caputo T.M., Cusano A.M., Ruvo M., Aliberti A., Cusano A. (2022). Human Serum Albumin Nanoparticles as a Carrier for On-Demand Sorafenib Delivery. Curr. Pharm. Biotechnol..

[71] Paz-Ares L., Hirsh V., Zhang L., de Marinis F., Yang J.C., Wakelee H.A., Seto T., Wu Y.L., Novello S., Juhász E. (2015). Monotherapy Administration of Sorafenib in Patients With Non-Small Cell Lung Cancer (MISSION) Trial: A Phase III, Multicenter, Placebo-Controlled Trial of Sorafenib in Patients with Relapsed or Refractory Predominantly Nonsquamous Non-Small-Cell Lung Cancer after 2 or 3 Previous Treatment Regimens. J. Thorac. Oncol..

[72] Zheng N., Liu W., Li B., Nie H., Liu J., Cheng Y., Wang J., Dong H., Jia L. (2019). Co-delivery of sorafenib and metapristone encapsulated by CXCR4-targeted PLGA-PEG nanoparticles overcomes hepatocellular carcinoma resistance to sorafenib. J. Exp. Clin. Cancer Res..

[73] Zhong T., Liu X., Li H., Zhang J. (2021). Co-delivery of sorafenib and crizotinib encapsulated with polymeric nanoparticles for the treatment of. Drug Deliv..

[74] Taha A.M., Aboulwafa M.M., Zedan H., Helmy O.M. (2022). Ramucirumab combination with sorafenib enhances the inhibitory effect of sorafenib on HepG2 cancer cells. Sci. Rep..

[75] Hung W.H., Zheng J.H., Lee K.C., Cho E.C. (2019). Doxorubicin conjugated AuNP/biopolymer composites facilitate cell cycle regulation and exhibit superior tumor suppression potential in KRAS mutant colorectal cancer. J. Biotechnol..

[76] Wang L., Liu X., Zhang C., Chen X., Sheng W., Li P., Qin D., Wang F. (2023). Novel amphiphilic hydroxyethyl starch-based nanoparticles loading camptothecin exhibit high anticancer activity in HepG2 cells and zebrafish. Colloids Surf. B Biointerfaces.

[77] Kong C., Li Y., Liu Z., Ye J., Wang Z., Zhang L., Kong W., Liu H., Liu C., Pang H. (2019). Targeting the Oncogene KRAS Mutant Pancreatic Cancer by Synergistic Blocking of Lysosomal Acidification and Rapid Drug Release. ACS Nano.

[78] Thummuri D., Khan S., Underwood P.W., Zhang P., Wiegand J., Zhang X., Budamagunta V., Sobh A., Tagmount A., Loguinov A. (2022). Overcoming Gemcitabine Resistance in Pancreatic Cancer Using the BCL-X. Mol. Cancer Ther..

[79] Fanchon L.M., Russell J., Pillarsetty N., O’Donoghue I., Gangangari K., Yu K.H., Humm J.L. (2020). Comparing the intra-tumoral distribution of Gemcitabine, 5-Fluorouracil, and Capecitabine in a murine model of pancreatic ductal adenocarcinoma. PLoS ONE.

[80] Das M., Li J., Bao M., Huang L. (2020). Nano-delivery of Gemcitabine Derivative as a Therapeutic Strategy in a Desmoplastic KRAS Mutant Pancreatic Cancer. AAPS J..

[81] Liu X., Jiang J., Liao Y.P., Tang I., Zheng E., Qiu W., Lin M., Wang X., Ji Y., Mei K.C. (2021). Combination Chemo-Immunotherapy for Pancreatic Cancer Using the Immunogenic Effects of an Irinotecan Silicasome Nanocarrier Plus Anti-PD-1. Adv. Sci..

[82] Liu X., Jiang J., Chang C.H., Liao Y.P., Lodico J.J., Tang I., Zheng E., Qiu W., Lin M., Wang X. (2021). Development of Facile and Versatile Platinum Drug Delivering Silicasome Nanocarriers for Efficient Pancreatic Cancer Chemo-Immunotherapy. Small.

[83] Kesik-Brodacka M. (2018). Progress in biopharmaceutical development. Biotechnol. Appl. Biochem..

[84] Moorkens E., Meuwissen N., Huys I., Declerck P., Vulto A.G., Simoens S. (2017). The Market of Biopharmaceutical Medicines: A Snapshot of a Diverse Industrial Landscape. Front. Pharmacol..

[85] Lagassé H.A., Alexaki A., Simhadri V.L., Katagiri N.H., Jankowski W., Sauna Z.E., Kimchi-Sarfaty C. (2017). Recent advances in (therapeutic protein) drug development. F1000Res.

[86] Wahlich J., Desai A., Greco F., Hill K., Jones A.T., Mrsny R.J., Pasut G., Perrie Y., Seib F.P., Seymour L.W. (2019). Nanomedicines for the Delivery of Biologics. Pharmaceutics.

[87] Rafael D., Montero S., Carcavilla P., Andrade F., German-Cortés J., Diaz-Riascos Z.V., Seras-Franzoso J., Llaguno M., Fernández B., Pereira A. (2023). Intracellular Delivery of Anti-Kirsten Rat Sarcoma Antibodies Mediated by Polymeric Micelles Exerts Strong. ACS Appl. Mater. Interfaces.

[88] Sakamoto K., Qi Y., Miyako E. (2023). Nanoformulation of the K-Ras(G12D)-inhibitory peptide KS-58 suppresses colorectal and pancreatic cancer-derived tumors. Sci. Rep..

[89] Singh S., Murillo G., Richner J., Singh S.P., Berleth E., Kumar V., Mehta R., Ramiya V., Parihar A.S. (2022). A Broad-Based Characterization of a Cell-Penetrating, Single Domain Camelid Bi-Specific Antibody Monomer That Targets STAT3 and KRAS Dependent Cancers. Int. J. Mol. Sci..

[90] Haza K.Z., Martin H.L., Rao A., Turner A.L., Saunders S.E., Petersen B., Tiede C., Tipping K., Tang A.A., Ajayi M. (2021). RAS-inhibiting biologics identify and probe druggable pockets including an SII-α3 allosteric site. Nat. Commun..

[91] Röth S., Macartney T.J., Konopacka A., Chan K.H., Zhou H., Queisser M.A., Sapkota G.P. (2020). Targeting Endogenous K-RAS for Degradation through the Affinity-Directed Protein Missile System. Cell Chem. Biol..

[92] Ajmal A., Ali Y., Khan A., Wadood A., Rehman A.U. (2022). Identification of novel peptide inhibitors for the KRas-G12C variant to prevent oncogenic signaling. J. Biomol. Struct. Dyn..

[93] Samad A., Khurshid B., Mahmood A., Rehman A.U., Khalid A., Abdalla A.N., Algarni A.S., Wadood A. (2023). Identification of novel peptide inhibitors for oncogenic KRAS G12D as therapeutic options using mutagenesis-based remodeling and MD simulations. J. Biomol. Struct. Dyn..

[94] Alonso Fernandez M.J., Desireé T.O. (2020). Intracellular Delivery of Anti-KRAS Antibodies Formulated into Nanocapsules. https://patents.google.com/patent/EP3962491A1/en.

[95] Bio L. (2022). Enabling Intracellular Antibody Delivery to Unlock High-Value Targets. https://www.nature.com/articles/d43747-022-00073-x.

[96] Pei Y., Chen L., Huang Y., Wang J., Feng J., Xu M., Chen Y., Song Q., Jiang G., Gu X. (2019). Sequential Targeting TGF-β Signaling and KRAS Mutation Increases Therapeutic Efficacy in Pancreatic Cancer. Small.

[97] Anthiya S., Öztürk S.C., Yanik H., Tavukcuoglu E., Şahin A., Datta D., Charisse K., Álvarez D.M., Loza M.I., Calvo A. (2023). Targeted siRNA lipid nanoparticles for the treatment of KRAS-mutant tumors. J. Control. Release.

[98] Maghsoudnia N., Eftekhari R.B., Sohi A.N., Dorkoosh F.A. (2021). Chloroquine Assisted Delivery of microRNA Mimic Let-7b to NSCLC Cell Line by PAMAM (G5) - HA Nano-Carrier. Curr. Drug Deliv..

[99] Shahidi M., Abazari O., Dayati P., Haghiralsadat B.F., Oroojalian F., Tofighi D. (2022). Targeted delivery of 5-fluorouracil, miR-532-3p, and si-KRAS to the colorectal tumor using layer-by-layer liposomes. Front. Bioeng. Biotechnol..

[100] Xue W., Dahlman J.E., Tammela T., Khan O.F., Sood S., Dave A., Cai W., Chirino L.M., Yang G.R., Bronson R. (2014). Small RNA combination therapy for lung cancer. Proc. Natl. Acad. Sci. USA.

[101] Pecot C.V., Wu S.Y., Bellister S., Filant J., Rupaimoole R., Hisamatsu T., Bhattacharya R., Maharaj A., Azam S., Rodriguez-Aguayo C. (2014). Therapeutic silencing of KRAS using systemically delivered siRNAs. Mol. Cancer Ther..

[102] Kim M.J., Lee S.J., Ryu J.H., Kim S.H., Kwon I.C., Roberts T.M. (2020). Combination of KRAS gene silencing and PI3K inhibition for ovarian cancer treatment. J. Control. Release.

[103] O’Brien Z., Wang L., Majeti B., Clamme J., Baclig R., Chu J., Fong S., Harborth J., Ibarra J., Yin H. A novel lipid nanoparticle (NBF-006) encapsulating glutathione S-transferase P (GSTP) siRNA for the treatment of KRAS-driven non-small cell lung cancer. Proceedings of the AACR Annual Meeting.

[104] Cecchin R., Troyer Z., Witwer K., Morris K.V. (2023). Extracellular vesicles: The next generation in gene therapy delivery. Mol. Ther..

[105] Mendt M., Kamerkar S., Sugimoto H., McAndrews K.M., Wu C.C., Gagea M., Yang S., Blanko E.V.R., Peng Q., Ma X. (2018). Generation and testing of clinical-grade exosomes for pancreatic cancer. JCI Insight.

[106] Kamerkar S., LeBleu V.S., Sugimoto H., Yang S., Ruivo C.F., Melo S.A., Lee J.J., Kalluri R. (2017). Exosomes facilitate therapeutic targeting of oncogenic KRAS in pancreatic cancer. Nature.

[107] Mehta A., Dalle Vedove E., Isert L., Merkel O.M. (2019). Targeting KRAS Mutant Lung Cancer Cells with siRNA-Loaded Bovine Serum Albumin Nanoparticles. Pharm. Res..

[108] Strand M.S., Krasnick B.A., Pan H., Zhang X., Bi Y., Brooks C., Wetzel C., Sankpal N., Fleming T., Goedegebuure S.P. (2019). Precision delivery of RAS-inhibiting siRNA to KRAS driven cancer via peptide-based nanoparticles. Oncotarget.

[109] Pekow J., Meckel K., Dougherty U., Butun F., Mustafi R., Lim J., Crofton C., Chen X., Joseph L., Bissonnette M. (2015). Tumor suppressors miR-143 and miR-145 and predicted target proteins API5, ERK5, K-RAS, and IRS-1 are differentially expressed in proximal and distal colon. Am. J. Physiol. Gastrointest. Liver Physiol..

[110] Yoshikawa Y., Taniguchi K., Tsujino T., Heishima K., Inamoto T., Takai T., Minami K., Azuma H., Miyata K., Hayashi K. (2019). Anti-cancer Effects of a Chemically Modified miR-143 on Bladder Cancer by Either Systemic or Intravesical Treatment. Mol. Ther. Methods Clin. Dev..

[111] Takai T., Tsujino T., Yoshikawa Y., Inamoto T., Sugito N., Kuranaga Y., Heishima K., Soga T., Hayashi K., Miyata K. (2019). Synthetic miR-143 Exhibited an Anti-Cancer Effect via the Downregulation of K-RAS Networks of Renal Cell Cancer Cells In Vitro and In Vivo. Mol. Ther..

[112] Wan T., Chen Y., Pan Q., Xu X., Kang Y., Gao X., Huang F., Wu C., Ping Y. (2020). Genome editing of mutant KRAS through supramolecular polymer-mediated delivery of Cas9 ribonucleoprotein for colorectal cancer therapy. J. Control Release.

[113] Liu C., Zhang L., Liu H., Cheng K. (2017). Delivery strategies of the CRISPR-Cas9 gene-editing system for therapeutic applications. J. Control. Release.

[114] Wan T., Pan Q., Liu C., Guo J., Li B., Yan X., Cheng Y., Ping Y. (2021). A Duplex CRISPR-Cas9 Ribonucleoprotein Nanomedicine for Colorectal Cancer Gene Therapy. Nano Lett..

[115] McAndrews K.M., Xiao F., Chronopoulos A., LeBleu V.S., Kugeratski F.G., Kalluri R. (2021). Exosome-mediated delivery of CRISPR/Cas9 for targeting of oncogenic Kras. Life Sci. Alliance.

[116] Gupta R., Vishwakarma L., Guleri S.K., Kumar G. (2022). 5-Fluorouracil-Impregnated PLGA Coated Gold Nanoparticles for Augmented Delivery to Lung Cancer: In Vitro Investigations. Anticancer Agents Med. Chem..

[117] McDaid W.J., Greene M.K., Johnston M.C., Pollheimer E., Smyth P., McLaughlin K., Van Schaeybroeck S., Straubinger R.M., Longley D.B., Scott C.J. (2019). Repurposing of Cetuximab in antibody-directed chemotherapy-loaded nanoparticles in EGFR therapy-resistant pancreatic tumours. Nanoscale.

[118] Zhang C., Gao F., Wu W., Qiu W.X., Zhang L., Li R., Zhuang Z.N., Yu W., Cheng H., Zhang X.Z. (2019). Enzyme-Driven Membrane-Targeted Chimeric Peptide for Enhanced Tumor Photodynamic Immunotherapy. ACS Nano.

[119] Roncato F., Rruga F., Porcù E., Casarin E., Ronca R., Maccarinelli F., Realdon N., Basso G., Alon R., Viola G. (2018). Improvement and extension of anti-EGFR targeting in breast cancer therapy by integration with the Avidin-Nucleic-Acid-Nano-Assemblies. Nat. Commun..

[120] Luo D., Xu X., Iqbal M.Z., Zhao Q., Zhao R., Farheen J., Zhang Q., Zhang P., Kong X. (2021). siRNA-Loaded Hydroxyapatite Nanoparticles for *KRAS* Gene Silencing in Anti-Pancreatic Cancer Therapy. Pharmaceutics.

[121] Zhang X., Lin Z.I., Yang J., Liu G.L., Hu Z., Huang H., Li X., Liu Q., Ma M., Xu Z. (2021). Carbon Dioxide-Derived Biodegradable and Cationic Polycarbonates as a New siRNA Carrier for Gene Therapy in Pancreatic Cancer. Nanomaterials.

[122] Sreedurgalakshmi K., Srikar R., Harikrishnan K., Srinivasan L., Rajkumari R. (2021). Cetuximab-siRNA Conjugate Linked Through Cationized Gelatin Knocks Down KRAS G12C Mutation in NSCLC Sensitizing the Cells Toward Gefitinib. Technol. Cancer Res. Treat..

[123] Wu L.P., Wang D., Li Z. (2020). Grand challenges in nanomedicine. Mater. Sci. Eng. C Mater. Biol. Appl..

[124] Yang C., Merlin D. (2023). Challenges to Safe Nanomedicine Treatment. Nanomaterials.

[125] Metselaar J.M., Lammers T. (2020). Challenges in nanomedicine clinical translation. Drug Deliv. Transl. Res..

